# Therapeutic effect of bismuth subsalicylate in a propionic acid–induced autism model

**DOI:** 10.1007/s00210-025-04255-z

**Published:** 2025-05-15

**Authors:** Kubilay Doğan Kılıç, Burak Çakar, Yiğit Uyanıkgil, Lora Koenhemsi, Berzah Güneş, Ebru Eroğlu, Oytun Erbaş

**Affiliations:** 1 Department of Histology and Embryology, Faculty of Medicine, Ege University, İzmir, Türkiye; 2 Museum Für Naturkunde, Leibniz Institute for Evolution and Biodiversity Science, Berlin, Germany; 3 Department of Histology and Embryology, Faculty of Medicine, Istinye University, Istanbul, Türkiye; 4 Department of Stem Cell, Institute of Health Sciences, Ege University, İzmir, Türkiye; 5 Department of Internal Medicine, Faculty of Veterinary Medicine, Istanbul University-Cerrahpasa, Istanbul, Türkiye; 6 Institute of Experimental Medicine, Istanbul, Türkiye; 7 Faculty of Medicine, BAMER, Biruni University, Istanbul, Türkiye

**Keywords:** Autism, Neuroinflammation, Oxidative stress, Bismuth subsalicylate, Propionic acid

## Abstract

Inflammation-induced oxidative stress in macrophages and microglia is associated with excessive production of reactive oxygen species, initiating a damaging cycle of neuroinflammation and cellular injury. These processes are significant contributors to the pathophysiology of autism spectrum disorders, which involve neuronal dysfunction, cell loss, and behavioral impairments. Under conditions of oxidative stress, activated microglia release pro-inflammatory mediators, further intensifying neuronal damage. Bismuth subsalicylate (BSS), a compound with well-documented anti-inflammatory and antioxidant properties, has shown potential in mitigating such neurodegenerative processes. This study aimed to evaluate the effects of BSS in reducing neuroinflammation and oxidative stress in a propionic acid (PPA)-induced autism model, alongside its impact on behavioral outcomes. The study utilized 30 male Wistar albino rats, with PPA administered intraperitoneally at 250 mg/kg/day for 5 days to induce an autism-like phenotype. Rats were divided into three groups: Group 1 (Normal control, *n* = 10); Group 2 (PPA + saline, PPAS, *n* = 10); and Group 3 (PPA + BSS, PPAB, *n* = 10). Treatments were administered for 15 days. Behavioral performance was assessed through three-chamber sociability, open field, and passive avoidance learning tests, followed by biochemical and histological evaluations of brain tissues. Biochemical analysis revealed a significant increase in malondialdehyde, tumor necrosis factor-alpha, and interleukin-17 levels in the PPAS group, indicating heightened oxidative stress and inflammation. Treatment notably reduced these markers, suggesting its efficacy in mitigating oxidative damage and inflammatory responses. Immunohistochemical results demonstrated reduced glial activation and enhanced neuronal preservation in the hippocampal and cerebellar regions of treated rats. Additionally, behavioral impairments in social interaction, exploration, and memory were significantly improved with BSS therapy. These results suggest that BSS may confer neuroprotective effects through attenuation of oxidative stress and neuroinflammation, potentially contributing to improved neuronal function and behavioral performance in a PPA-induced autism model.

## Introduction

Autism spectrum disorder (ASD) is a neurodevelopmental condition marked by social communication challenges and repetitive behaviors. Although the pathophysiology of ASD remains poorly understood, oxidative stress and neuroinflammation are recognized as central mechanisms contributing to its progression (Usui et al. 2023; Zawadzka et al. 2021). These processes often lead to neuronal damage, reduced neuroplasticity, and behavioral deficits, all of which are hallmarks of ASD pathology.

Oxidative stress, driven by an overproduction of reactive oxygen species (ROS), is commonly observed in individuals with ASD. This imbalance in oxidative homeostasis has been associated with lipid, mitochondrial dysfunction, and an increase in malondialdehyde (MDA), a key marker of oxidative damage (de Mattos et al. 2020). The resulting oxidative stress may contribute to chronic neuroinflammation, as evidenced by elevated pro-inflammatory cytokines such as TNF-α (Carlson et al. 1999), IL-6 (di Penta et al. 2013), IL-17 (MacMahon Copas et al. 2024), and prostaglandin E2 (PGE2) (Jiang et al. 2019). These inflammatory mediators also reduce neurotrophic factors like brain-derived neurotrophic factor (BDNF), which are essential for neuronal survival and synaptic plasticity (Kerschensteiner et al. 1999; Lai et al. 2018).

Bismuth subsalicylate (BSS), a widely used anti-inflammatory compound, has recently garnered attention for its potential gastrointestinal and neuroprotective properties (Abdou et al. 2022; Borbinha et al. 2019; Figueroa-Quintanilla et al. 1993; Sox and Olson 1989; Steinhoff et al. 1980). BSS has been shown to modulate oxidative stress by reducing lipid peroxidation and scavenging free radicals (Bagchi et al. 1999; Tillman et al. 1996) while also inhibiting inflammatory pathways mediated by cytokines like IL-6 (di Penta et al. 2013), TNF-α (Carlson et al. 1999), and PGE2 (Jiang et al. 2019). By suppressing PGE2 levels, BSS may alleviate neuroinflammatory responses and protect neuronal integrity. Its ability to cross the blood–brain barrier and directly target neuroinflammatory processes makes it a promising candidate for addressing the pathological mechanisms underlying ASD (Borbinha et al. 2019; Reynolds et al. 2012).

This study investigated the effects of BSS on oxidative stress, neuroinflammation, and behavioral abnormalities in a propionic acid (PPA)-induced rat model of ASD. The PPA model mimics key features of ASD, including increased oxidative stress, elevated pro-inflammatory cytokine levels (Alsubaiei et al. 2023; Bin-Khattaf et al. 2022; Choi et al. 2018), and deficits in social and cognitive behaviors (Bin-Khattaf et al. 2022; Choi et al. 2018, 2018). The propionic acid (PPA)-induced autism model has been extensively validated in the literature for reproducing core features of autism spectrum disorder (ASD), including social deficits, repetitive behaviors, neuroinflammation, oxidative stress, and mitochondrial dysfunction. Previous studies by Macfabe et al. (2007), Bhandari and Kuhad (2017), and Lobzhanidze et al. (2020) have consistently demonstrated the reliability and translational relevance of this model for ASD research (Bhandari and Kuhad 2017; Macfabe et al. 2007; Lobzhanidze et al. 2020). By evaluating the impact of BSS on these parameters, this research aims to provide novel insights into its therapeutic potential for managing ASD symptoms.

## Materials and methods

### Animals

In this study, 30 male Wistar albino rats, weighing 150-200 g and 10-12 week old, were used. The experiments performed in this study have been carried out according to the rules in the Guide for the Care and Use of Laboratory Animals adopted by National Institutes of Health (U.S.A), practically. Having received Animal Ethics Committee’s consent (Demiroglu Bilim University, Ethical number: 0923032808).

All animal procedures were conducted in compliance with the institutional animal care guidelines and the EU Directive 2010/63/EU, legally.

To minimize distress, animals were habituated to handling and behavioral test environments prior to experimentation. Behavioral testing was carried out in a quiet, dimly lit room. Handling was limited to trained personnel using gentle, consistent techniques. No painful or invasive procedures were performed, and animals were monitored daily for signs of distress.

The rats used in the experiment were obtained from Experimental Animal laboratory of Demioglu Bilim University. Rats were fed ad libitum and housed impairs in steel cages having a temperature-controlled environment (22 ± 2 °C) with 12-h light/dark cycles.

### Experimental procedures

The experimental design involved an autism model using 30 male Wistar albino rats. Propionic acid (PPA, CheMondis GmbH, CAS: 79–09-4, Köln, Germany) was used to induce autism-like behaviors. Twenty rats underwent PPA induction via intraperitoneal injections of 250 mg/kg/day for five consecutive days. Ten rats served as the normal control group without PPA exposure.

The rats were randomly divided into three groups as follows (Tiwari et al. 2021):

- Group 1 (Control, *n* = 10): Received only saline orally and served as the normal control group.
- Group 2 (PPA + Saline, PPAS, *n* = 10): Induced with PPA and treated with 1 ml/kg/day of 0.9% NaCl saline by oral gavage.
- Group 3 (PPA + BSS, PPAB, *n* = 10): Induced with PPA and treated with 60 mg/kg/day BSS (Spectrum Chemical, SPC-B1703-25GM, Novato, CA 94949 USA) by oral gavage.

All treatments were administered for 15 days. Behavioral assessments, including the three-chamber sociability test, open field test, and passive avoidance learning test, were conducted between 10:00 AM and 3:00 PM following the treatment period.

The BSS dose was selected based on prior preclinical research demonstrating its efficacy in reducing oxidative stress and neuroinflammation in rodent models. Effective doses were scaled appropriately for the rat model, in alignment with previous studies reporting neuroprotective and anti-inflammatory effects of BSS at 60 mg/kg (Pitz et al. 2015; Bowen et al. 2019; Boyarkin et al. 2024).

At the end of the study, rats were euthanized under anesthesia using ketamine (100 mg/kg, Ketasol, Richter Pharma AG, Austria) and xylazine (50 mg/kg, Rompun, Bayer, Germany). Blood samples were collected by cardiac puncture for biochemical analyses, and brain tissues were stored at − 80 °C for histological evaluations.

### Behavioral tests

#### Three-chamber sociability test

**Fig. 1:**
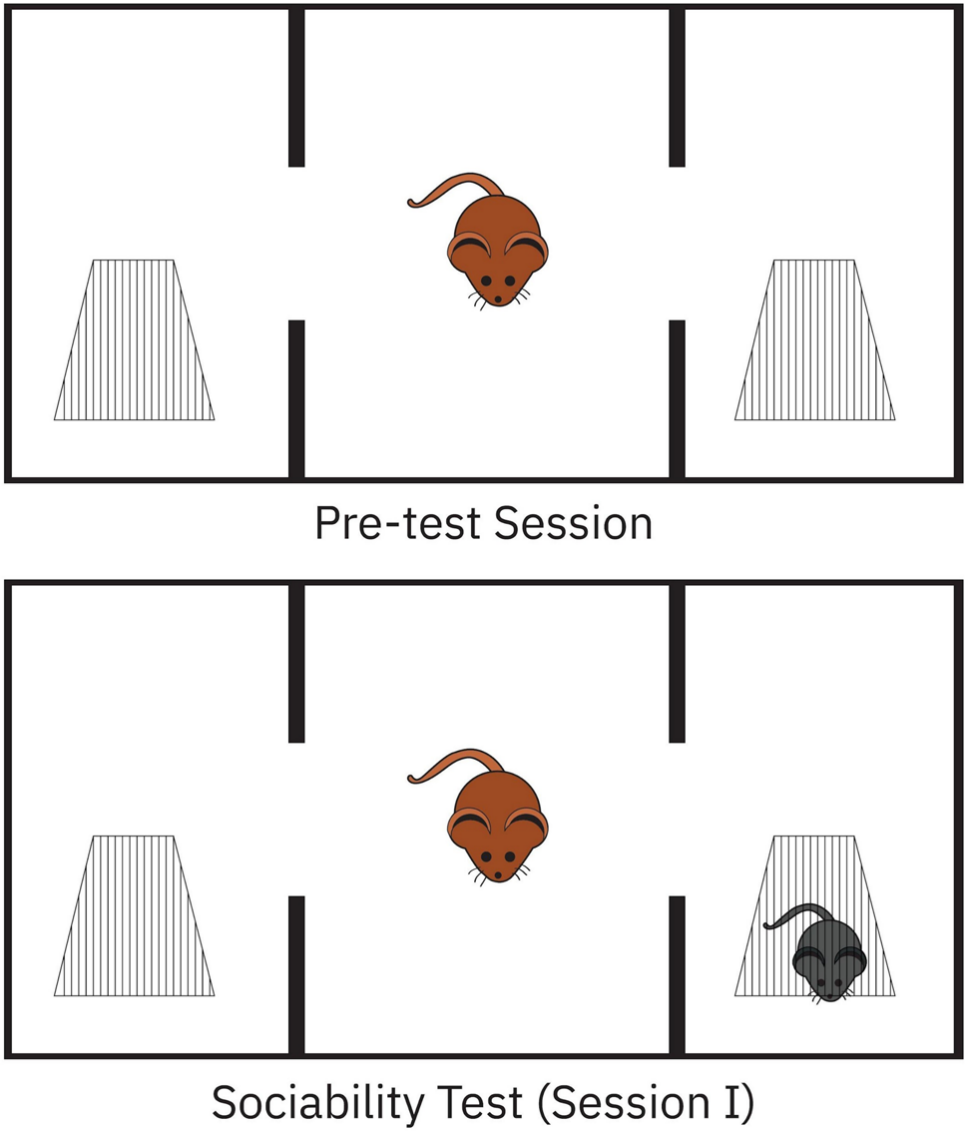
Demonstration of pre-test session and sociability test with three-chamber sociability test

Sociability test was performed as previously described with minor modifications (Erbas et al. 2018). Briefly, a Plexiglas cage (40 × 90 × 40 cm) was divided into three equal regions (40 × 30 × 40 cm). On the first day, the rats were allowed to habituate in the test cage for 5 min (pre-test session). Twenty-four hours later, to test sociability, a stranger rat (Stranger 1) was placed inside a small plastic cage with mesh-like holes in one side chamber and an empty cage in the third chamber. Then, the test rat was placed in the center chamber, and the time spent in each region by the test rat was recorded for 10 min (Session I). The test rat was in the chamber when its head and two front paws entered the chamber. The floor of the field was then cleaned between each test with a 70% alcohol and dried with paper towel to remove any traces of olfactory stimuli from the previous rat (Fig. 1). Time spent with Stranger calculated as a percentage.

#### Open-field test

The open-field (OF) paradigm is one of the popular behavioral tests to assess locomotion and exploration. Altered OF behavior is relatively simple to observe; however, concluding the reasons for the observed changes is a complex task. Generally, there are two factors that determine the behavior in this paradigm: The first is a positive exploratory drive originating from the nature of rodents to explore new environments (for food and shelter), and the second is the animal nature of avoiding open and brightly lit spaces (exposure to predators). The open-field test is considered useful in determining motor stereotyped behavior, repeated autogrooming, and restriction of research activity in autism model (Erbas et al. 2018). The open-field test was conducted in an open-aired box with dimensions of 50 × 50 × 40 cm. At the beginning of the test, rats were gently placed in the center of the box and allowed to explore the arena freely for 5 min. Then, each rat was observed for 5 min to evaluate its spontaneous activity level. The total number of ambulation (i.e., the number of floor divisions crossed with four paws) was recorded. The floor of the field was then cleaned between each animal with a 70% alcohol-water solution and dried with paper towel to avoid olfactory cues.

#### Passive avoidance learning (PAL)

Learning and memory performance of offspring were evaluated by passive avoidance learning (PAL) test as FCA described previously (Erbas et al. 2018). PAL is comprised of fear-motivated avoidance tasks in which the rat learns to refrain from stepping through a door that seems apparently safer but actually leads into a dark compartment with an electrified grid system that delivers a shock. The PAL box was 20 × 20 × 20 cm and had both dark and lighted chambers. Normally, when a rat was placed into the lighted compartment, they preferred to enter the dark chamber. After a 10-s habituation period in the lighted compartment, the guillotine door separating the light and dark chambers was opened. When a rat passed into the dark chamber, the door separating the light and dark compartments was closed. Then, a 1.5-mA electric shock was delivered over 3 s, and the rat was subsequently removed from the dark chamber and returned to its cage. Twenty-four hours later, the rats were placed into the PAL box again. The duration of time or latency period for the rat to travel from the lighted to the dark chamber was recorded, but a shock was not delivered. The latency period was recorded up to a maximum of 300 s. The time that the rat took to refrain from crossing into the dark chamber served as an index of the rat’s memory.

#### Immunohistochemistry

The Cornu Ammonis (CA) 1 and CA 3 regions of hippocampus and cerebellum were chosen as the target areas to be examined for hippocampus damage. Briefly, following behavioral tests, animals were euthanized and their brains removed and fixed 3 days in 10% formalin (Sigma-Aldrich, HT501128, Massachusetts, USA) in 0.1 M phosphate-buffer saline (PBS) (Sigma-Aldrich, P4417-100 TAB, Massachusetts, USA). Then, they were moved into 30% sucrose (ISOLAB, CAS 57–50-1, Türkiye) and stored at 4 °C until infiltration was complete. The brains were cut coronally on a sliding microtome at 40 µm and mounted on gelatinized glass slides (LabScientific, CE-7802–1, Massachusetts, USA). For glial fibrillar acidic protein (GFAP) immunohistochemistry (Abcam, ab7260, Inc., MA, US; 1/1000), brain sections were incubated with 10% H_2_O2 for 30 min to eliminate endogenous peroxidase activity and blocked with 10% normal goat serum (Invitrogen, cas 31872, USA) for 1 h at room temperature. Subsequently, sections were incubated in primary antibodies against GFAP for 24 h at 4 °C. Antibody detection was performed with the Histostain-Plus Bulk kit (Invitrogen, cat 85–8943, USA) against rabbit IgG, and 3,3′-diaminobenzidine (DAB) (Abcam, ab64238, Cambridge, UK) was used to visualize the final product. All sections were washed in PBS and photographed with an Olympus C5050 digital camera mounted on microscope (Olympus Corporation, Olympus BX51, Japan).

To calculate the GFAP immunostaining index, GFAP-positive cells were counted at × 40 magnification in randomized Sects. (3–4) for each rat. All histopathological examinations were performed by the same investigators who were blinded to the study groups and each other. This procedure was performed with an image analysis system (Image-Pro Express 1.4.5, Media Cybernetics, Inc. USA) in four sections per studied group.

Cresyl violet staining (Abcam, ab246816, USA) to quantify the number of surviving neuron counts was performed in six sections per studied group by an image same analysis system.

#### Biochemical analysis

After decapitation, brains were rapidly removed and stored at − 20 °C until biochemical analysis. For tissue analysis, whole cerebral tissues were homogenized with a glass homogenizer in 5% volume of PBS (pH, 7.4) and centrifuged (Thermo Scientific/Micro CL17R, USA) at 5000 g for 15 min. The supernatant was then collected, and total protein concentration in the brain homogenates was determined according to Bradford’s method using bovine serum albumin as standard (Bradford 1976).

The brain levels of TNF-α (Abcam, ab220210, USA), BDNF (Abcam, ab226843, USA), IL-17 (Abcam, ab214588, USA), PGE2 (Abcam, ab124419, USA), and IL-6 (Abcam, ab6672, USA) in the brain supernatants were measured using commercially available rat enzyme-linked immunosorbent assay (ELISA) kits. All samples from each animal were measured in duplicate according to the manufacturer’s guidelines. A microplate reader was used for the measurement of the Absorbances (MultiscanGo, Thermo Fisher Scientific Laboratory Equipment, NH, USA).

Lipid peroxidation was determined in brain tissue samples by measuring malondialdehyde (MDA) (Abcam, ab27642, USA) levels as thiobarbituric acid reactive substances (TBARS). Briefly, trichloroacetic acid and TBARS reagent were added to the brain tissue samples, then mixed and incubated at 100 °C for 60 min. After cooling on ice, the samples were centrifuged at 3000 rpm for 20 min, and the absorbance of the supernatant was read at 535 nm. MDA levels were calculated from the standard calibration curve using tetraethoxypropane and expressed as nanomoles per gram protein.

### Statistical analysis

Statistical evaluation was performed using SPSS version 15.0 for Windows (SPSS Inc., Chicago, IL, USA). Following one-way ANOVA, Bonferroni correction was applied to all post-hoc multiple comparisons to control for type I error. Shapiro–Wilk’s *W* and Levene’s tests were used to check the normality and the homogeneity of variance, respectively. The results are presented as mean ± standard error of the mean (SEM). The value of *p* < 0.05 was accepted as statistically significant.

Post-hoc power analyses were conducted across behavioral, biochemical, and histological data using G*Power. For PAL test, TNF-α levels, and CA1 neuronal counts, calculated effect sizes yielded statistical power values of 1.00 (*α* = 0.05), confirming the adequacy of the sample size (*n* = 10 per group). Sample size was determined based on standard practices in PPA-induced ASD models.

## Results

### Behavioral tests

#### Three-chamber sociability test

The three-chamber sociability test assessed the ability of rats to engage in social interaction. Rats in the PPA + saline (PPAS) group spent significantly less time interacting with the unfamiliar rat (30.1 ± 1.9%) compared to the normal control group (70.5 ± 3.8%). One-way ANOVA identified significant differences across the groups. Post-hoc analyses confirmed a marked decrease in sociability in the PPAS group relative to controls.

In contrast, rats in the PPA + BSS (PPAB) group exhibited an increased percentage of time spent interacting with the unfamiliar rat (68.9 ± 5.2%), which was significantly higher than the PPAS group. These results suggest that BSS treatment mitigated the social impairments induced by PPA exposure, restoring sociability levels comparable to those of the control group (Table 1).

**Table 1:** Behavioral test results

	Normal control	PPAS	PPAB
*Sociability test*: *The spend of time with stranger rat percent (%)*	70.5 ± 3.8	30.1 ± 1.9 **	68.9 ± 5.2 ##
*Open-field test: Number of ambulation*	16.9 ± 4.05	3.7 ± 1.6 **	8.4 ± 1.1 #
*Passive avoidance learning (PAL) Latency (Sec.)*	261.7 ± 23.8	57.9 ± 18.2 **	228.5 ± 14.7 ##

Results were presented as mean ± SEM. Statistical analyses were performed by one-way ANOVA. *p < 0.01, **p < 0.001: different from normal groups; #*p* < 0.05, ##*p* < 0.001: different from PPA and saline group. *p < 0.05, **p < 0.001: significantly different from normal control group; #*p* < 0.05, ##*p* < 0.001: significantly different from PPA + saline group. Statistical significance was determined using one-way ANOVA followed by Bonferroni post-hoc test

#### Open-field test

The open field test was utilized to evaluate exploratory behavior and anxiety levels in the rats. The number of crossings in the open field was significantly reduced in the PPAS group (3.7 ± 1.6) compared to the normal control group (16.9 ± 4.05). One-way ANOVA revealed significant group differences. Post-hoc analyses showed a marked reduction in exploratory behavior in the PPAS group compared to the control group.

Conversely, rats in the PPAB group demonstrated a significant improvement in the number of crossings (8.4 ± 1.1), which was notably higher than that observed in the PPAS group. These findings indicate that BSS treatment enhanced exploratory activity and reduced anxiety-like behavior. These results suggest that BSS may have anxiolytic properties, promoting more normal exploratory behavior in the PPA-induced autism model.

#### Passive avoidance learning

The passive avoidance test was conducted to evaluate the learning and memory abilities of the rats. The latency to avoid the dark compartment was significantly shorter in the PPAS group (57.9 ± 18.2 s) compared to the normal control group (261.7 ± 23.8 s), as determined by one-way ANOVA. Post-hoc analysis revealed a significant difference between the PPAS group and the control group, indicating substantial impairment in learning and memory caused by PPA exposure.

In contrast, the PPAB group demonstrated a significantly longer latency to avoid the dark compartment (228.5 ± 14.7 s) compared to the PPAS. These results suggests that BSS treatment improved learning and memory abilities in rats exposed to PPA and also indicates that BSS has a neuroprotective effect, counteracting the cognitive deficits induced by PPA. A comprehensive evaluation of biochemical, histological, and behavioral outcomes supports the therapeutic potential of BSS in reducing oxidative stress and neuroinflammation, preserving neuronal integrity, and enhancing cognitive and behavioral performance in a PPA-induced autism model.

#### Immunohistochemical analysis

**Fig. 2:**
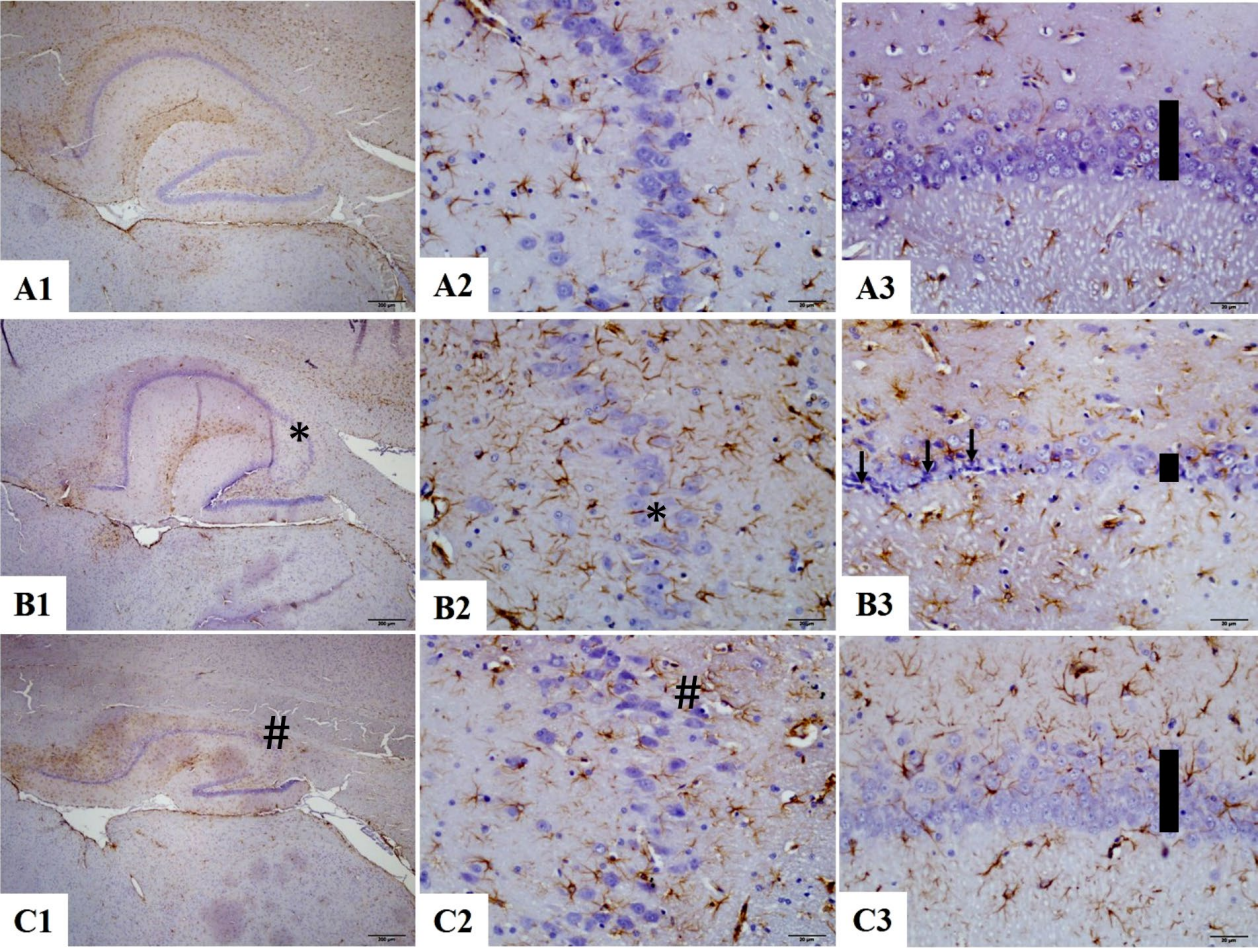
CA3 and CA1 of hippocampus × 40 magnification. Astrogliosis was characterized by GFAP immunostaining (Brown staining). **A1**–**A3** Normal control group male rats CA3 and CA1. **B1**–**B3** PPA and saline group male rats have increased glial activity and decreased neural count and dysmorphological changes CA3 and CA1 pyramidal neuron (arrow and asterisk) (*p < 0.05 vs Control). **C1**–**C3** PPA and BSS group male rats have decreased glial activity and increased count and improved neural morphology changes CA3 and CA1 (hash) pyramidal neuron (#*p* < 0.05 vs PPA group). Vertical bars showing the thickness in selected demonstrative parts. Statistical significance symbols are overlaid on the images. Quantitative data are presented in corresponding bar graphs. Statistical analysis: one-way ANOVA with Bonferroni post-hoc test

GFAP immunostaining was performed to assess astrocyte activation and glial activity in the hippocampus and cerebellum. In the hippocampal CA1 and CA3 regions, astrogliosis was prominent in the PPAS group, as indicated by a significant increase in GFAP-positive staining. In the CA1 region, the PPAS group displayed elevated glial activity (50.8 ± 1.9) compared to the normal control group (33.8 ± 3.5). Treatment with BSS (PPAB group) significantly attenuated this increase (42.02 ± 2.2). Similarly, in the CA3 region, the PPAS group exhibited heightened GFAP expression (45.2 ± 1.4) compared to the control group (31.6 ± 1.7), while the PPAB group showed reduced levels of GFAP (36.7 ± 1.9) (Table 2, Fig. 2).

**Table 2:** Adjusted cell counting results

	Normal control	PPAS	PPAB
*Neuronal Count CA1*	71.5 ± 3.7	55.5 ± 2.4 **	65.1 ± 0.9 #
*Neuronal Count CA3*	43.7 ± 1.1	29.4 ± 1.6 **	40.1 ± 1.5 ##
*GFAP immunostaining index (CA1)*	33.8 ± 3.5	50.8 ± 1.9 *	42.02 ± 2.2 #
*GFAP immunostaining index (CA3)*	31.6 ± 1.7	45.2 ± 1.4 *	36.7 ± 1.9 #
*Purkinje count cerebellum*	25.6 ± 0.9	13.8 ± 1.3 *	19.8 ± 0.7 #
*GFAP immunostaining index (cerebellum)*	19.5 ± 1.9	30.6 ± 2.1 *	23.1 ± 1.6 #

. Results were presented as mean ± SEM. Statistical analyses were performed by one-way ANOVA. *p < 0.05, **p < 0.001: different from normal groups; #*p* < 0.05, ##*p* < 0.001: different from PPA and saline group. *p < 0.05, **p < 0.001: significantly different from normal control group; #*p* < 0.05, ##*p* < 0.001: significantly different from PPA + saline group. Statistical significance was determined using one-way ANOVA followed by Bonferroni post-hoc test

Neuronal integrity was evaluated based on neuronal counts in the CA1 and CA3 regions. The PPAS group demonstrated a significant reduction in neuronal numbers in the CA1 region (55.5 ± 2.4) compared to the control group (71.5 ± 3.7). The PPAB group exhibited a partial recovery in neuronal counts (65.1 ± 0.9). Similarly, in the CA3 region, neuronal counts were significantly lower in the PPAS group (29.4 ± 1.6) than in the control group (43.7 ± 1.1), but BSS treatment improved neuronal survival (40.1 ± 1.5) (Table 2).

**Fig. 3:**
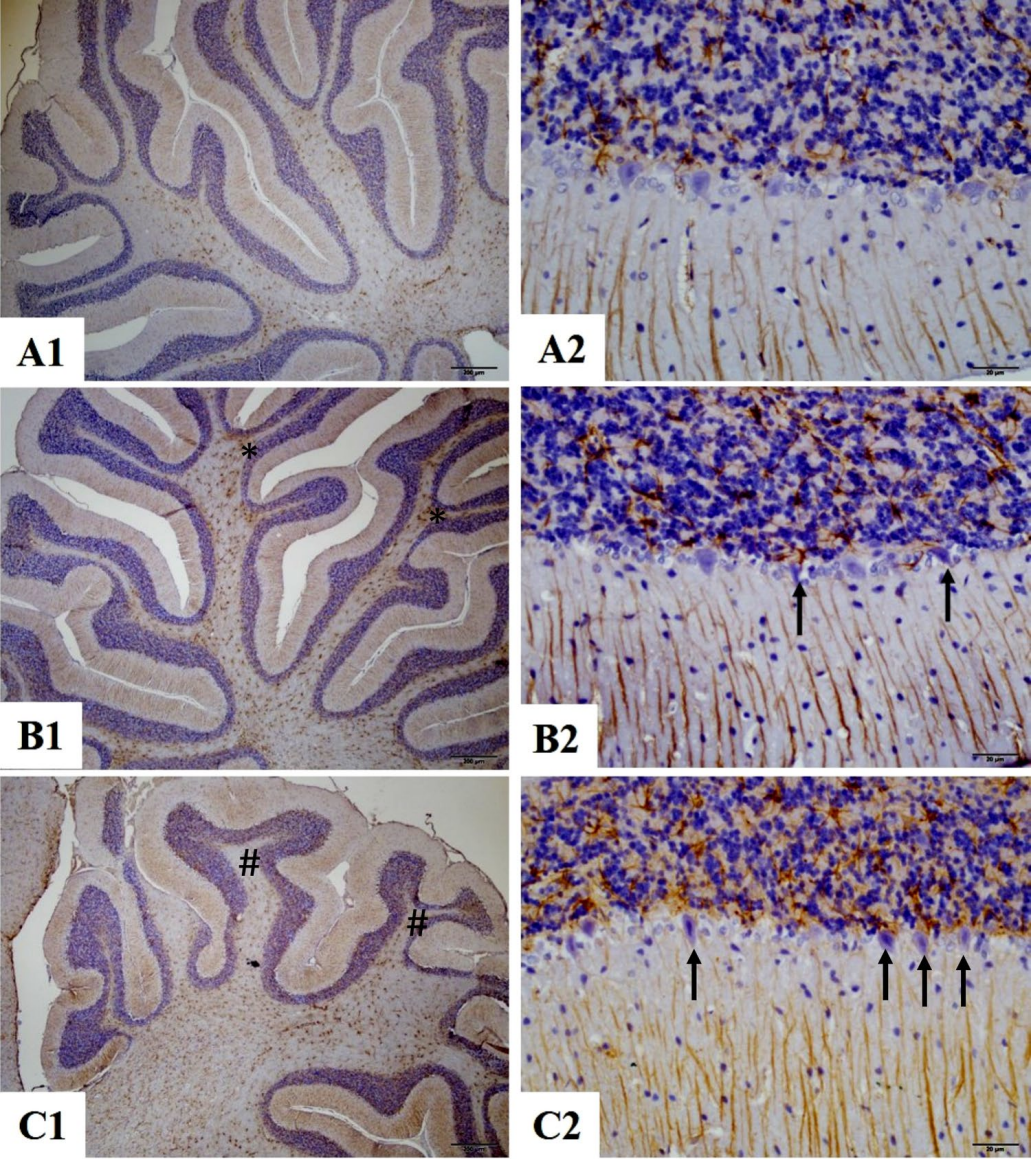
Cerebellum × 40 magnification. Astrogliosis was characterized by GFAP immunostaining (Brown staining). **A1**, **A2** Normal control group male rats. **B1**, **B2** PPA and saline group male rats have increased glial activity and decreased and dysmorphological changes in cerebellum Purkinje Neuron (arrow and asterisk) (*p < 0.05 vs Control). **C1**, **C2** PPA and BSS group male rats have decreased glial activity and increased count and improved Purkinje Neuron morphology changes in cerebellum (arrow and hash) structure (#*p* < 0.05 vs PPA group). Statistical markers are shown on image panels. Data were analyzed using one-way ANOVA followed by Bonferroni correction

In the cerebellum, GFAP immunostaining revealed increased glial activation in the PPAS group (30.6 ± 2.1) compared to the normal control group (19.5 ± 1.9). The PPAB group showed a reduction in GFAP-positive staining (23.1 ± 1.6). Purkinje cell counts were also significantly reduced in the PPAS group (13.8 ± 1.3) compared to the control group (25.6 ± 0.9). BSS treatment significantly preserved Purkinje cell integrity in the PPAB group (19.8 ± 0.7) (Table 2, Fig. 3).

These findings indicate that PPA exposure induces substantial astrocyte activation, gliosis, and neuronal loss in both hippocampal and cerebellar regions. BSS treatment effectively mitigates these pathological changes, likely through its anti-inflammatory and antioxidant properties, preserving neuronal and glial integrity.

### Biochemical analysis

Biochemical analysis revealed significant changes in oxidative stress markers, inflammatory cytokines, and neurotrophic factors among the experimental groups, underscoring the neuroprotective effects of BSS (Table 3).

**Table 3:** Biochemical analyses results

	Normal control	PPA + saline	PPA + BSS
*Brain MDA level (nmol/gr protein)*	53.3 ± 1.8	158.2 ± 7.3 **	67.1 ± 4.3 ##
*Brain IL-6 level (pg/mg protein)*	185.3 ± 11.8	309.5 ± 10.9 *	218.6 ± 13.1 #
*Brain TNF-alfa level (pg/mg protein)*	17.5 ± 1.6	161.5 ± 12.8 **	115.2 ± 7.5 #
*Brain IL-17 level (pg/mg protein)*	163.2 ± 10.9	465.8 ± 9.3 **	208.7 ± 6.3 ##
*Brain PGE2 level (pg/mg protein)*	17.8 ± 0.8	32.4 ± 4.4 *	21.5 ± 1.7 #
*Brain BDNF level (pg/mg protein)*	67.5 ± 5.09	39.1 ± 6.5 *	50.3 ± 3.9 #

Results were presented as mean ± SEM. Statistical analyses were performed by one-way ANOVA. *p < 0.05, **p < 0.001: different from normal groups; #*p* < 0.05, ##*p* < 0.001: different from PPA and saline group. *p < 0.05, **p < 0.001: significantly different from normal control group; #*p* < 0.05, ##*p* < 0.001: significantly different from PPA + saline group. Statistical significance was determined using one-way ANOVA followed by Bonferroni post-hoc test

MDA levels, an oxidative stress parameter, were markedly elevated in the PPA + saline (PPAS) group (158.2 ± 7.3 nmol/gr protein) compared to the normal control group (53.3 ± 1.8). Treatment with BSS significantly reduced MDA levels in the PPAB group (67.1 ± 4.3), highlighting its antioxidant capacity in mitigating oxidative damage.

Inflammatory cytokines were notably increased following PPA exposure. IL-6 levels were significantly higher in the PPAS group (309.5 ± 10.9 pg/mg protein) compared to the control group (185.3 ± 11.8). Treatment with BSS significantly decreased IL-6 levels in the PPAB group (218.6 ± 13.1). Similarly, TNF-α levels, a critical pro-inflammatory cytokine, were elevated in the PPAS group (161.5 ± 12.8 pg/mg protein) compared to the control group (17.5 ± 1.6). BSS treatment significantly reduced TNF-α levels in the PPAB group (115.2 ± 7.5).

IL-17 and PGE2, both involved in inflammation, followed a similar pattern. IL-17 levels were significantly elevated in the PPAS group (465.8 ± 9.3 pg/mg protein) compared to controls (163.2 ± 10.9), while the PPAB group showed significant reductions (208.7 ± 6.3). Likewise, PGE2 levels were higher in the PPAS group (32.4 ± 4.4 pg/mg protein) compared to controls (17.8 ± 0.8), and BSS treatment significantly decreased PGE2 levels (21.5 ± 1.7).

BDNF levels, a neurotrophic factor vital for neuronal health and plasticity, were significantly reduced in the PPAS group (39.1 ± 6.5 pg/mg protein) compared to the control group (67.5 ± 5.09). BSS treatment partially restored BDNF levels in the PPAB group (50.3 ± 3.9), indicating a possible contribution to neuronal plasticity, though not a full restoration. Results are expressed as mean ± SEM, with statistical analyses performed using one-way ANOVA followed by post-hoc comparisons. Treatment’s statistical significance is denoted in Table 3**:** *p < 0.05, **p < 0.001 compared to the normal control group; #*p* < 0.05, ##*p* < 0.001 compared to the PPAS.

## Discussion

The present study explored the neuroprotective effects of BSS on oxidative stress, inflammation, neuronal integrity, and behavioral outcomes in a PPA-induced autism model in rats. The results indicate that BSS significantly reduced oxidative damage, mitigated inflammation, preserved neuronal integrity, and improved social and cognitive behaviors. Elevated MDA levels in the PPAS group confirmed the role of oxidative stress in the PPA-induced autism model, consistent with previous studies reporting increased oxidative stress markers in ASD (Alfawaz et al. 2022; Erbas et al. 2018; Kamalmaz et al. 2023; MacFabe et al. 2008; Mirza and Sharma 2018, 2019). BSS treatment significantly reduced MDA levels in the PPAB group, highlighting its potent antioxidant properties. The ability of BSS to reduce lipid peroxidation and scavenge free radicals suggests that BSS may be effective in reducing oxidative stress, which has been implicated as a pathological factor in ASD.

Pro-inflammatory cytokines such as TNF-α, IL-6, IL-17, and PGE2 were significantly elevated in the PPAS group, indicating a robust inflammatory response following PPA administration (Alam 2021; Alvarez et al. 2020; Beringer et al. 2017; Haase et al. 2021; Jovanovic et al. 1998; Kany et al. 2019; Ore et al. 2024; Saghazadeh et al. 2019; Wautier and Wautier 2023). Elevated PGE2 levels, in particular, emphasize the role of prostaglandins in amplifying neuroinflammation and disrupting neuronal signaling pathways (Milatovic et al. 2011; Zhu et al. 2020). BSS administration significantly reduced the levels of these cytokines in the PPAB group, demonstrating its anti-inflammatory effects. BSS is known to modulate inflammatory pathways by suppressing the activation of nuclear factor kappa B (NF-κB) (Cavallini et al. 2001; Kopp and Ghosh 1994) and other pro-inflammatory signaling molecules (Chu et al. 2016; Yang et al. 2020), which likely contributes to its neuroprotective effects observed in this study (Cavallini et al. 2001). These findings align with the well-established connection between neuroinflammation and autism-like behaviors in animal models (Bronzuoli et al. 2018; Kazlauskas et al. 2015).

The preservation of neuronal integrity in the hippocampal CA1 and CA3 regions further supports the neuroprotective role of BSS. The PPAB group exhibited a significant reduction in neuronal loss compared to the PPAS group, suggesting that BSS protects against PPA-induced neurodegeneration (Sajid et al. 2023). This observation aligns with previous studies demonstrating the neuroprotective effects of anti-inflammatory compounds in neurodegenerative models; BSS’s antioxidant (Buga et al. 2024; Lim et al. 2024) and anti-inflammatory (Li et al. 2024) properties likely contribute to its ability to counteract oxidative stress and inflammation, thereby preserving neuronal survival (Bagchi et al. 1999; Usui et al. 2023) and plasticity (Abdou et al. 2022; Lai et al. 2018).

Behavioral assessments revealed significant improvements in the PPAB group compared to the PPAS group. In the three-chamber sociability test, rats treated with BSS spent more time interacting with an unfamiliar rat, indicating improved sociability (Sahin et al. 2022), which is a core deficit in ASD (Özkul et al. 2022). In the open field test, the PPAB group exhibited increased exploratory activity and reduced anxiety-like behavior, suggesting a potential anxiolytic effect of BSS (Tiwari et al. 2021). Similarly, the passive avoidance learning (PAL) test showed improved learning and memory in the PPAB group, further supporting the cognitive benefits of BSS (Vinogradov et al. 2009). These behavioral improvements are likely a result of its neuroprotective (Buga et al. 2024) and anti-inflammatory (Jiang et al. 2019) effects, which help preserve (Ghosh et al. 1994) and synaptic plasticity (Leal et al. 2015).

Another notable finding was the partial restoration of BDNF levels in the PPAB group. BDNF is crucial for neuronal survival (Ghosh et al. 1994), growth (Azman and Zakaria 2022), and synaptic plasticity (Leal et al. 2015), and its deficiency has been linked to neurodegenerative disorders and cognitive impairments (Amidfar et al. 2020; Azman and Zakaria 2022; Mitre et al. 2017). The increase in BDNF levels following BSS treatment suggests that BSS may enhance neuroplasticity and cognitive function (Quialheiro et al. 2022; Vinogradov et al. 2009), critical for alleviating ASD symptoms (Kamalmaz et al. 2023).

These findings contribute to a growing body of evidence supporting the therapeutic potential of BSS in neurodevelopmental and neuroinflammatory conditions. BSS employs a multifaceted approach to neuroprotection by reducing oxidative stress (di Penta et al. 2013), suppressing inflammation (Bagchi et al. 1999), and promoting neurotrophic support (Lai et al. 2018). This study highlights the potential of BSS as a therapeutic agent for ASD, providing a novel approach to targeting key pathological mechanisms underlying the disorder. Future research should focus on the long-term effects of BSS and its potential clinical applications, particularly in dietary or pharmacological interventions for managing ASD symptoms.

Recent studies have identified a range of genetic and epigenetic contributors to ASD, including mutations in genes such as SHANK3 and MECP2, as well as altered DNA methylation patterns (Ciernia and LaSalle 2016; Moessner et al. 2007). These mechanisms influence synaptic structure, gene expression, and neurodevelopmental timing. Moreover, several alternative treatments have shown promise in modulating oxidative stress and inflammation, such as sulforaphane, bumetanide, and resveratrol (Al-Beltagi et al. 2024; Uchino and Waga 2013). While BSS targets overlapping pathways, its dual anti-inflammatory and antioxidant properties may offer complementary therapeutic benefits.

Although BSS is an FDA-approved compound for gastrointestinal indications, its long-term safety and tolerability in the context of neurodevelopmental disorders remain uncertain. Potential side effects include gastrointestinal discomfort, constipation, and, with prolonged use, the risk of bismuth accumulation and neurotoxicity. Moreover, the translational applicability of our findings is limited by species differences in metabolism, dosage sensitivity, and blood–brain barrier permeability. Therefore, further preclinical and clinical studies are essential to establish the feasibility of BSS as a therapeutic candidate for ASD. Another limitation of the present study is the absence of behavioral assessments targeting repetitive behaviors and sensory sensitivities, which are core symptoms of ASD. Future studies should incorporate marble-burying and ultrasonic vocalization paradigms to provide a more comprehensive behavioral profile.

Due to the absence of pathway-specific or mechanistic assays, causal relationships between oxidative stress reduction and behavioral outcomes cannot be definitively established. Future studies should employ molecular inhibitors or mechanistic probes to clarify these interactions.

## Conclusion

This study explored the neuroprotective potential of BSS in a PPA-induced autism model in rats. The findings demonstrated that BSS significantly attenuated oxidative stress, as indicated by reduced MDA levels, and mitigated neuroinflammation through the suppression of pro-inflammatory cytokines such as TNF-α and IL-6. These results highlight the potent antioxidant and anti-inflammatory properties of BSS, which contribute to its neuroprotective effects by preserving neuronal integrity and partially restoring BDNF levels.

Behavioral analyses further revealed that BSS treatment improved sociability, exploratory activity, and cognitive function, suggesting its potential to alleviate core autism-like symptoms, including social interaction deficits and cognitive impairments.

Overall, this study provides compelling evidence that BSS could serve as a promising therapeutic agent for ASD by targeting crucial pathological mechanisms, including oxidative stress, inflammation, and neuronal damage. Future studies should aim to investigate the long-term efficacy and clinical applicability of BSS to further establish its role in ASD treatment.

## Data Availability

The datasets used and/or analysed during the current study available from the corresponding author on reasonable request.
